# Emergence of Daptomycin Nonsusceptibility and Treatment Failure in Patients With *Corynebacterium striatum* Bacteremia

**DOI:** 10.1093/ofid/ofae610

**Published:** 2024-10-10

**Authors:** Shunkichi Ikegaki, Goh Ohji, Kei Furui Ebisawa, Mitsutaka Tsujimura, Kenichiro Ohnuma, Kentaro Iwata

**Affiliations:** Division of Infectious Disease Therapeutics, Department of Microbiology and Infectious Diseases, Kobe University Graduate School of Medicine, Kobe, Japan; Division of Infectious Disease Therapeutics, Department of Microbiology and Infectious Diseases, Kobe University Graduate School of Medicine, Kobe, Japan; Division of Infectious Disease Therapeutics, Department of Microbiology and Infectious Diseases, Kobe University Graduate School of Medicine, Kobe, Japan; Division of Infectious Disease Therapeutics, Department of Microbiology and Infectious Diseases, Kobe University Graduate School of Medicine, Kobe, Japan; Department of Clinical Laboratory, Kobe University Hospital, Kobe, Japan; Division of Infectious Disease Therapeutics, Department of Microbiology and Infectious Diseases, Kobe University Graduate School of Medicine, Kobe, Japan

**Keywords:** *Corynebacterium*, daptomycin, resistance, susceptibility, vancomycin

## Abstract

We retrospectively reviewed patients with *Corynebacterium striatum* bacteremia treated with daptomycin. All 11 isolates were initially susceptible to daptomycin, but the emergence of daptomycin nonsusceptibility during treatment and clinical failure occurred in 36% and 45% of patients, respectively.

*Corynebacterium striatum* is a gram-positive, rod-shaped bacterium that colonizes normal human skin and mucosal membranes. It has been increasingly recognized as a significant nosocomial pathogen, particularly in patients who are immunocompromised. Although vancomycin is the most frequently used antibiotic for *C striatum* infection [1], it is not universally tolerated because of its toxicity. Daptomycin is a cyclic lipopeptide antibiotic with broad-spectrum activity against gram-positive bacteria and is used as an alternative to vancomycin for gram-positive bacterial infections. *C striatum* has a high susceptibility rate for daptomycin [2, 3], and successful treatment has been reported [4, 5]. However, in recent years, there has been an increasing number of case reports of high-level daptomycin resistance in *C striatum* associated with daptomycin exposure [6–10]. This phenomenon has also been observed in vitro at a high frequency [11, 12]. The frequency of the emergence of nonsusceptibility in patients who received daptomycin therapy remains unclear. In this retrospective study, we aimed to determine the rate of clinical failure and the emergence of daptomycin nonsusceptibility in patients with *C striatum* bacteremia.

## METHODS

### Study Population

This retrospective study was conducted with the medical record data of Kobe University Hospital from 1 January 2012 to 20 February 2024. Kobe University Hospital is a referral hospital in Honshu Island, Japan, with 934 beds. At this hospital, vancomycin is essentially the drug of choice for treating *C striatum* bacteremia. Daptomycin is an alternative drug that requires preauthorization by infectious disease physicians. We identified all adult patients with positive blood cultures of *C striatum* during the study period. Patients were included in the study if (1) *C striatum* was judged to be a true cause of bacteremia, not a contaminant; (2) daptomycin was administered to the patient for at least 1 day as a treatment for *C striatum* bacteremia; and (3) daptomycin susceptibility of the *C striatum* isolate was tested. Each case was reviewed by multiple infectious disease physicians to distinguish between true bacteremia and contamination. Demographic information, clinical diagnosis, treatment course, and laboratory data were extracted from the patients' medical records. The study protocol was approved by the Ethics Committee of Kobe University Hospital.

### Identification and Susceptibility Testing

Identification of *C striatum* was performed with either MALDI Biotyper (MBT reference library version 4, 9, or 12; Bruker) or BD BBL Crystal Gram Positive ID System (Becton Dickinson). Susceptibility to daptomycin was tested with either Etest (bioMérieux) or the broth microdilution method with dry-plate Eiken. Susceptibility testing for daptomycin was routinely reported after September 2023; before that, it was reported and stored in the medical records at the physician's request.

### Outcomes

We determined the emergence of daptomycin nonsusceptibility as the primary outcome. Emergence of nonsusceptibility was defined as the initial isolate being susceptible and any isolate from a specimen obtained during treatment or within 30 days after treatment cessation being nonsusceptible to daptomycin. Susceptibility was determined by the breakpoint in document M45 of the Clinical and Laboratory Standards Institute [13]. As the institute defines only the breakpoint for susceptibility, we used the term “nonsusceptible” for minimum inhibitory concentration (MIC) values >1 μg/mL. We also evaluated the rate of clinical failure as a secondary outcome, which was defined as the deterioration or nonimprovement of symptoms, vital signs, or laboratory data that could not be explained by the progression of diseases other than *C striatum* infection occurring during or after daptomycin treatment. Clinical failure was judged by the agreement of at least 2 infectious disease physicians.

### Statistical Analysis

Descriptive statistics were used to report the patient characteristics and outcomes of the study. The nonsusceptibility rate was defined as the number of patients who developed daptomycin nonsusceptibility divided by the number of all included patients. The clinical failure rate was defined as the number of patients with clinical failure divided by the total number of included patients.

## RESULTS

*C striatum* was identified in 122 adult patients during the study period. Susceptibility to daptomycin was tested in 18 patients. The initial isolates from these patients were susceptible to daptomycin, except for 1 isolate with prior daptomycin administration. Of these, 11 patients were administered daptomycin as a treatment of *C striatum* bacteremia, with 7 receiving daptomycin for >7 days. All 11 cases were confirmed as true bacteremia through a chart review by the infectious disease physicians. Details of these patients are summarized in Table 1. Nonsusceptibility emerged in 4 of the 11 patients (36%), and clinical failure occurred in 5 of the 11 patients (45%). Among the patients with clinical failure, 4 had been treated with daptomycin for >7 days, while 1 had received daptomycin for only a single day. Patients with clinical failure were diagnosed with either febrile neutropenia or infective endocarditis. Patient 5 developed persistent *C striatum* bacteremia during antibiotic treatment for infective endocarditis caused by *Enterococcus faecalis*. The patient finally underwent valve replacement surgery, and culture of the valve tissue revealed *C striatum*. Patients 10 and 11 were clinically diagnosed with catheter-related bloodstream infections. Indwelling catheters were retained in patient 10 but removed in patient 11. Patient 11 had an artificial aortic graft, but no signs of graft infection were observed. Patients 3, 4, and 6 had ureteral stents that were replaced for treatment in all patients.

**Table 1.** Clinical Characteristics and Outcomes of the Patients

No.	Age, y	Sex	Background Disease	Clinical Diagnosis	Clinical Failure	Duration of DAP, d	Initial Dose of DAP, mg/kg	Initial MIC, μg/mL^a^	Subsequent Culture	Subsequent MIC, μg/mL	Emergence of NS	Interval to Become NS, d
1	76	F	Acute myeloid leukemia	FN	Yes	24	5.8	≤0.5	Positive	>4	Yes	39
2	57	F	Acute myeloid leukemia	FN	Yes	15	5.9	≤0.5	Positive	>4	Yes	27
3	56	F	Uterine cancer	cUTI	No	11	6.7	≤0.5	Negative	…	No	…
4	63	F	Uterine cancer	cUTI	No	7	6.7	≤0.5	Not performed	…	No	…
5	76	M	IE^b^	IE	Yes	7	5.9	≤0.5	Positive	>4	Yes	4
6	81	M	Bladder cancer	cUTI	No	23	10.3	≤0.5	Negative	…	No	…
7	67	M	Non-Hodgkin lymphoma	FN	Yes	1	5.6	≤0.25	Positive	≤0.25	No	…
8	56	F	Acute myeloid leukemia	FN	No	4	6.4	≤0.25	Negative	…	No	…
9	73	M	Acute myeloid leukemia	FN	No	3	6.0	≤0.25	Negative	…	No	…
10	65	M	Liver transplantation	CRBSI	No	4	6.3	≤0.25	Negative	…	No	…
11	82	F	Aortic dissection	CRBSI, IE	Yes	12	6.0	≤0.25	Positive	>4	Yes	3

Abbreviations: CRBSI, catheter-related bloodstream infection; cUTI, complicated urinary tract infection; DAP, daptomycin; F, female; FN, febrile neutropenia; IE, infective endocarditis; M, male; MIC, minimum inhibitory concentration; NS, nonsusceptibility.

^a^All MICs were measured with dry-plate Eiken.

^b^IE caused by *Enterococcus faecalis*.

The initial dose of daptomycin was 5.6 to 10.3 mg/kg (median, 6.0 mg/kg). None of the patients had a body mass index >30. The duration between the initial blood culture and subsequent culture, in which nonsusceptibility was confirmed, ranged from 3 to 39 days.

## DISCUSSION

In this retrospective study, we confirmed that *C striatum* frequently became nonsusceptible to daptomycin during treatment, leading to clinical failure. Nonsusceptibility developed in as little as 3 days, which is consistent with previous in vitro observations that *C striatum* isolates can develop high-level resistance after brief exposure to daptomycin [11, 12]. To the best of our knowledge, this is the first study to evaluate the incidence of this phenomenon in the clinical setting. In the present study, nonsusceptibility cases were diagnosed with febrile neutropenia or endocarditis. In the literature, nonsusceptibility cases have also been reported in left ventricular assist device infections [7–9], intra-abdominal abscesses [10], and prosthetic joint infections [14], suggesting that daptomycin should be avoided, especially for complicated infections.

In our study population, the dose of daptomycin was at least 5.6 mg/kg. Although higher doses of daptomycin are associated with favorable outcomes for staphylococci and enterococci [15], it is unclear whether this applies to *C striatum.* It can express high-level daptomycin resistance through mutations in phosphatidylglycerol synthase (*pgsA2*), resulting in the loss of phosphatidylglycerol, a target molecule of daptomycin, from the membrane [16]. With this mechanism, the daptomycin MIC exceeds 256 μg/mL, a level that is unlikely to be overcome by a dose increase [17]. In our study as well as in previous in vitro studies, daptomycin resistance rapidly developed at a high frequency. It is more likely that preexisting *pgsA2* mutants were selected through daptomycin exposure rather than all the resistant strains having newly acquired mutations in *pgsA2*. The present study has several limitations. First, it was conducted at a single institution, which limits the external validity of our results. Second, because of the nature of rare infections, the number of patients enrolled in this study was small. Third, we could not confirm the emergence of high-level resistance because the MIC values for nonsusceptible isolates were above the measurement range of the broth microdilution plate. Nevertheless, considering the consistency of our findings with previous in vitro observations and case reports, treatment failure and the emergence of nonsusceptibility to daptomycin during treatment should be regarded as a common phenomenon, and daptomycin may not be a good treatment for treating *C striatum* bacteremia.

## Notes

***Acknowledgments***. We used Grammarly (https://www.grammarly.com) and Paperpal (https://paperpal.com) to edit our manuscript with regard to grammar and English usage.

***Author contributions*.** S. I. contributed to the study design, data analysis, interpretation of the results, and writing of the manuscript. G. O. and K. F. E. contributed to the design of the study and data analysis and reviewed the manuscript. M. T. contributed to data analysis and reviewed the manuscript. K. O. contributed to the acquisition and extraction of data, interpretation of results, and review of the manuscript. K. I. contributed to study design and manuscript review. All authors approved the final version of the manuscript.

***Patient consent statement*.** The study protocol was approved by the Ethics Committee of Kobe University Hospital.
